# Effects of Complex Training on Jumping and Change of Direction Performance, and Post-Activation Performance Enhancement Response in Basketball Players

**DOI:** 10.3390/sports11090181

**Published:** 2023-09-12

**Authors:** Piotr Biel, Paulina Ewertowska, Petr Stastny, Michał Krzysztofik

**Affiliations:** 1 Department of Sport and Physical Education, AGH University of Science and Technology, 30-059 Kraków, Poland; pbiel@agh.edu.pl; 2 Division of Clinical Physiotherapy, Faculty of Physical Education, Gdansk University of Physical Education and Sport, 80-336 Gdańsk, Poland; paulina.ewertowska@awf.gda.pl; 3 Department of Sport Games, Faculty of Physical Education and Sport, Charles University in Prague, 110 00 Prague, Czech Republic; stastny@ftvs.cuni.cz; 4 Institute of Sport Sciences, The Jerzy Kukuczka Academy of Physical Education, 40-065 Katowice, Poland

**Keywords:** sport performance, fatigue, exercise, post-activation performance enhancement, resistance training

## Abstract

Exercise order is one of the significant factors modulating training effects. Therefore, the aim of this study was to compare the effectiveness of an 8-week complex (CPX) training program utilizing intra-CPX active recovery with compound training (CMP) on bilateral and single-leg jumping performance, change of direction test time (shuttle test), and the post-activation performance enhancement (PAPE) response in a group of basketball players. Thirteen participants were performing CPX bi-weekly combined with regular pre-season basketball practice, while eleven participants were performing CMP for 8 weeks. Before and after the interventions, the following fitness tests were assessed: (i) bilateral countermovement jump, (ii) single-leg countermovement jump, (iii) shuttle run test. All tests were performed pre- and post-conditioning activity (CA—three sets of five drop jumps). The results showed a statistically significant increase in non-dominant (*p* = 0.019) and dominant single-leg jump relative peak power (*p* = 0.001), and in non-dominant single-leg jump height (*p* = 0.022) post-training compared to pre-training. The CA was significantly and similarly effective in eliciting a PAPE response in all tests before and after each intervention (*p* < 0.039; for all). However, the magnitude of improvement in CMJ and shuttle test time was trivial to small and did not reach statistical significance. Both 8 weeks of CPX and CMP training led to significant improvements in the SLJ power output of both the dominant and non-dominant limbs as well as the height of the non-dominant SLJ. Neither of the training methods had significant impacts on the magnitude of the PAPE response.

## 1. Introduction

The selection and order of exercises performed during a training session are among the moderators of the effectiveness of resistance training in eliciting improvements in athletic performance [1,2]. Both high-load, low-velocity resistance exercises and low-load, high-velocity exercises, such as plyometric or ballistic exercises, lead to improved athletic performance [3]. However, these adaptations occur in different ways. High-load resistance exercises primarily contribute to an increase in maximum and explosive strength, enhancing the force production component on the force–velocity curve [3]. Conversely, high-velocity exercises primarily allow for improvement in the velocity portion [3]. Therefore, employing both approaches in athlete preparation could allow for a wider range of adaptations.

The training strategy that combines these two types of exercises is called complex (CPX) training [4]. It involves performing exercises in pairs with a highly loaded resistance exercise that has a similar biomechanical movement structure to the subsequent high-velocity task [5]. For example, high-loaded squats before vertical jumps. This approach helps reap the benefits of both types of exercises. In contrast, it also allows for the elicitation of the post-activation performance enhancement (PAPE) effect, which results in a short-term improvement in physical fitness [6,7]. In the example mentioned, squats serve as the conditioning activity (CA) that immediately enhances performance in vertical jumps. The PAPE effect is generally observed around 5–7 min after performing low-volume CA (1–3 sets), involving high-resistance exercises (>85% one-repetition maximum [1 RM]) or high-velocity plyometric exercises [8,9]. This effect can be attributed to various mechanisms, including changes in muscle temperature, intramuscular fluid accumulation, neural mechanisms, or the phosphorylation of myosin regulatory light chains, as discussed by Blazevich and Babault [10].

These two aspects theoretically make CPX training superior to performing only resistance training or only plyometric training. Furthermore, considering the PAPE effect, performing a sequence of high-load exercises before high-velocity exercises may be more effective than performing them in a different manner (e.g., in blocks of plyometric or resistance exercises) [11,12,13]. Interestingly, studies comparing CPX training with other training strategies do not show statistically significant differences between approaches in improving athletic performance [12,14,15,16]. For example, Mihalik et al. [15] demonstrated similar improvements in power output and vertical jump height after 4 weeks of CPX training and compound training (consisting of one high-load resistance training session and one plyometric training session) conducted twice a week in a group of male and female volleyball players. In line with this, the results of a recent study by Gee et al. [12] showed no difference in the improvement of vertical jump performance, 10- and 40-m sprints, and the Arrowhead change of the direction time test between CPX training and reverse-contrast training (all plyometric exercises were done at the beginning of the training before high-load exercises) in a group of soccer players who trained twice a week for 10 weeks. Gee et al. [12] even suggested that, due to the need for long rest periods after the CA (4–8 min) during CPX training to achieve the PAPE effect, this training modality is not time-efficient. One idea for solving this problem is to add intra-CPX active recovery methods, such as focusing on a different part of the body [7,17,18]. This could allow for more training without affecting the PAPE effect [7]. To the best of the authors’ knowledge, there is only one study assessing the impact of intra-CPX active recovery on the PAPE effect [7], but no study has evaluated the long-term effects. 

Another aspect emphasized in the literature on PAPE, but not directly examined to the authors’ knowledge, is training experience and background [19]. These two aspects are indicated as significant factors modulating the magnitude of the PAPE response [19]. It is suggested that if an athlete has greater experience in resistance training, particularly with high loads and velocities, and a high level of muscular strength, they are more likely to take advantage of the PAPE effect [8,19,20]. Indeed, studies comparing the PAPE response clearly confirm that stronger individuals experience a greater PAPE effect [21]. However, it is unclear how adaptation occurs as a result of the long-term implementation of potentiation complexes, and whether it increases or diminishes.

To increase knowledge about the effectiveness of CPX training on athletic performance and the PAPE response, as well as to determine the impact of intra-CPX active recovery, the aim of this study was to compare the effectiveness of an 8-week CPX training program utilizing intra-CPX active recovery with compound training on bilateral and single-leg jumping performance, change of direction test time (shuttle test), and the PAPE response in a group of basketball players. The hypothesis was that both training protocols would significantly improve all test outcomes without any differences between them. Additionally, it was speculated that the PAPE response would significantly improve, but only in the group performing CPX training.

## 2. Materials and Methods

### 2.1. Experimental Approach to the Problem

A randomized, single-blind, parallel-group intervention was conducted to evaluate the effects of CPX training combined with regular basketball practice on bilateral and single-leg countermovement jump performance, shuttle test time, and PAPE effect response compared to traditional resistance training. The experimental group was performing CPX training bi-weekly combined with regular pre-season basketball practice, while the second group was performing CMP resistance training for 8 weeks. Before and after the interventions, the following fitness tests were assessed: (i) bilateral countermovement jump, (ii) single-leg countermovement jump, (iii) shuttle run test. All tests were performed pre- and post-CA (3 sets of 5 drop jumps).

### 2.2. Participants

**Figure 1:**
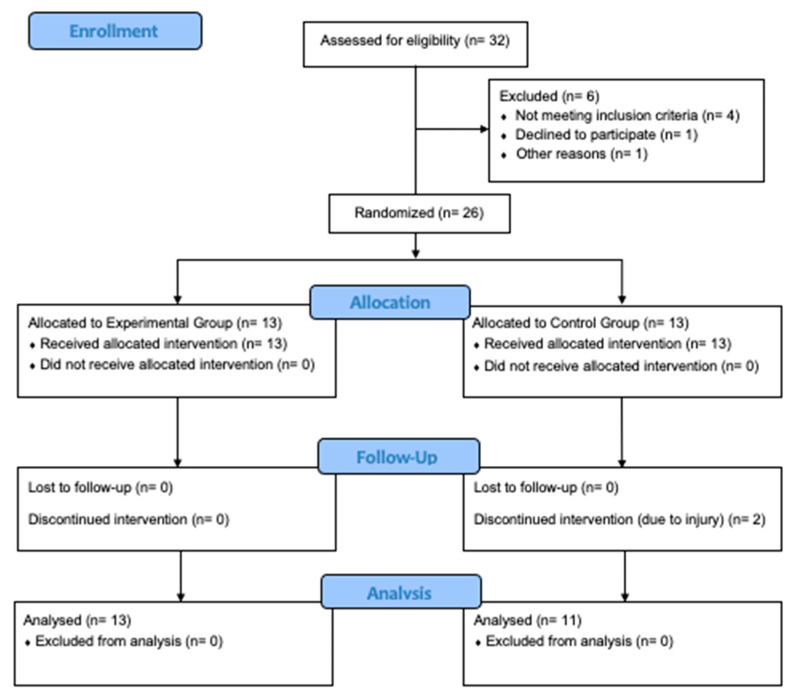
CONSORT flow diagram.

Thirty-two semi-professional basketball players were enrolled, but twenty-six of them met the eligibility criteria and were randomly allocated to the CPX or CMP groups (Figure 1). The participants were players from two competing teams in the second-tier level league of basketball in Poland. The inclusion criteria were: males aged 18–35; playing basketball at a semi-professional level for at least six years; engaged in regular basketball and resistance training at least twice a week for at least two years prior to the study; no history of serious injury or illness that affects basketball performance and that does not allow for full involvement in the training program. Finally, twenty-four participants completed the study (CPX: n = 13; age: 24 ± 6 yrs; body mass: 85.9 ± 10.9 kg; body height: 190.6 ± 7.9 cm; resistance training experience: 7 ± 5 yrs; basketball training experience: 12 ± 5 yrs; CMP: n = 11; age: 21 ± 4 yrs; body mass: 89.9 ± 8.5 kg; body height: 195.1 ± 10.3 cm; resistance training experience: 5 ± 4 yrs; basketball training experience: 11 ± 4 yrs). The intervention took part in the pre-season basketball practice, resulting in a training regimen that included two resistance training sessions and four basketball training sessions throughout the duration of this study. The participants were instructed not to change their lifestyles, including dietary habits, during the intervention period, and not to use any stimulants and alcoholic drinks throughout the study. Moreover, they were asked not to perform any additional resistance exercises 48-h before pre- and post-intervention evaluation to avoid the impact of fatigue on outcomes. Before giving their written informed consent for participation, subjects were informed about the advantages and potential risks of the investigation and provided with the option to withdraw from it at any time. Participants were not told of the expected study outcomes and the researchers involved in the measurements of outcomes were blinded. The study protocol was approved by the Bioethics Committee for Scientific Research, at the Academy of Physical Education in Katowice, Poland (3/2021) and performed according to the ethical standards of the Declaration of Helsinki 2013 [22]. The sample size was calculated a priori based on a statistical power of 0.8, an effect size of g = 0.31–0.48, and a significance level of 0.05, taking the effects of CPX training on vertical jumping and change of direction performance [16,23] as a reference variable. A minimum sample size of between 12–24 individuals was obtained (G*Power [version 3.1.9.2], Dusseldorf, Germany).

### 2.3. Procedures

#### 2.3.1. Familiarization Sessions

All sessions (familiarization, evaluation, and training) were conducted between 17:00 p.m. and 19:00 p.m. to prevent the impact of circadian rhythm on performance and preserve the regular training schedule. All sessions were preceded by a similar pretraining warm-up that included 5 min of jogging followed by exercises performed in a walk across the width of a basketball court (going back and forth for each exercise): glute walk, quad walk, high knees, knee hugs, and lunges (forward, backwards, and lateral). Then, in the same manner, as mentioned above, lateral jumping jacks, backpedaling, and ankle hops were performed. Finally, 2 circuits of 8–10 repetitions of the following exercises were performed: arm circles, forward and lateral leg swings, inchworms, and squats. In the first familiarization session (Monday), all participants performed 2 attempts of the CMJ, SLJ (for each leg), DJ, shuttle test, and isometric split squat (for each leg) to familiarize themselves with the study protocol. 

On the second familiarization session (Thursday), all participants took part in maximum strength evaluation in the back squat and bench press exercise to standardize training intensity during the upcoming training program. The testing was preceded by a standardized general warm-up, which consisted of riding on a cycling ergometer lasting approximately 5 min (with a resistance of ~100 W and cadence of ~70 rpm) and a dynamic part composed of body mass squats, arm circles, trunk rotations, side-bends, and push-ups for ten repetitions of each exercise. The 1 RM load for each exercise was indirectly calculated from the Epley formula [24]. Before the maximal attempt in each exercise, participants performed 3 sets with an estimated load of 50% of the estimated 1 RM for 15 repetitions followed by one set at 80% of the estimated 1 RM for 5 repetitions. Then, the load was increased to one that would allow performing a maximum of between 4 and 8 repetitions to calculate the 1 RM by the Epley formula. With this load, participants were instructed to perform as many repetitions as possible in order to produce a valid estimation of 1 RM [24].

$$1\mathrm{RM}=l\;\times (1+\frac{r}{30})$$

1 RM—one repetition maximum; l—load; r—number of performed repetitions.

In the end, all participants performed a single set of 4–10 repetitions of exercises that were scheduled to be performed in the upcoming training program (Table 1).

#### 2.3.2. Post Activation Performance Enhancement Evaluation Sessions

Two evaluation sessions were performed, 5–7 days before starting and 5–7 days after the end of the training program. After the same warm-up as in the first familiarization session, participants took part in CMJ, SLJ, and shuttle run baseline testing in random order. Two attempts for each test with 30 s rest were performed. Approximately 5 min later, participants performed a CA consisting of 3 sets of 5 DJs with a 60 s rest in-between. Then, after 6 min of passive rest, all tests were re-tested. DJs were executed from a 60 cm wooden box. To initiate the dropping action, participants were directed to step off the box one foot at a time and then rapidly jump upwards after ground contact, with emphasis on achieving maximum jump height. This instruction was designed to avoid jumping horizontally off the box. The selection of this CA was made based on prior research, which has shown its efficacy and simplicity in eliciting the PAPE effect [25,26].

#### 2.3.3. Training Program

The 8-week CPX training program is described in Table 1. This training was characterized by performing two sequences of exercises in each training session, one for the lower body and the other for the upper body. Each sequence consisted of CA as a resistance exercise (A1 or B1), then another exercise engaging different body regions as an active rest interval (A2 or B2), followed by an explosive exercise involving similar muscle groups as the first exercise (A3 or B3). The introduction of an active rest interval was dictated by the maintenance of a high training density.

Workouts were performed twice a week, on Monday (workout A) and Thursday (workout B), and on the rest of the weekdays, athletes participated in regular basketball practice. All workouts were supervised by the same strength and conditioning coach and were preceded by a standardized general warm-up, the same as during the second familiarization session. The CMP group performed the same exercises with the same volume and intensity but in a different order (explosive before resistance exercises), as presented in Table 2. 

#### 2.3.4. Measurement of Countermovement Jump Performance

The countermovement jumps performance was measured using force plates (Force Decks, Vald Performance^®^, Brisbane, QLD, Australia). This device has been previously confirmed as valid and reliable [27] for assessing vertical jump kinematics. The participant started in the standing position with hands placed on the hips. Then, they were instructed to perform a quick downward movement at a self-selected depth, followed by a fast-upward movement to jump as high as possible. The participant reset to the starting position after each jump, and the procedure was completed for a total of two jumps. The jump height, relative peak power, countermovement depth, and contraction time were evaluated. The best attempt in terms of jump height was retained for further evaluation. The jump height was calculated from the vertical velocity of the center of mass at take-off using the equation:

$$\mathrm{Jump}\;\mathrm{height}=\frac{1}{2}\cdot ({\mathrm{TOV}}^{2}/g)$$

where: TOV—vertical velocity of the center of mass at take-off; $g=9.81\;m\cdot s^{-2}$.

#### 2.3.5. Measurement of Shuttle Test Performance

The participants sprinted as quickly as possible linearly from the starting point for 12.5 m, touching a line on the ground with their foot, and then returning to the starting point after a 180° COD. Sprint times were recorded using timing photocells (SmartSpeed Pro, Fusion Sport, Coopers Plains, QLD, Australia), with gates at 0, 10, and 12.5 m. The height was set at approximately 1 m off the ground, corresponding to participants’ hip height to avoid the timing gates being triggered prematurely by a swinging arm or leg. The participants started with a front foot placed 0.3 m behind the first timing gate to prevent any early triggering of the photocells. Times were measured to the nearest 0.001 s. Two attempts were performed, and the best performance in terms of total COD time was retained for further analysis. 

## 3. Statistical Analysis

All data were analyzed using IBM SPSS Statistics for Macintosh, Version 25.0 (IBM Corp., Armonk, NY, USA) and were shown as means with standard deviations (±SD) with their 95% confidence intervals (CI). Statistical significance was set at *p* < 0.05. The normality of data distribution, assumption of variance homogeneity, and assumption of variance sphericity were verified using Shapiro–Wilk, Levene’s, and Mauchly’s tests, respectively. The repeated measures ANOVAs (2 [CPX; CMP] × 2 [baseline; post-training]) were used to investigate the influence of training programs on CMJ variables, shuttle test time, and percentage PAPE magnitude. Moreover, additional ANOVAs (2 [CPX; CMP] × 2 [pre-CA; post-CA] × 2 [baseline; post-training]) were used to assess PAPE response pre- and post-training. The effect sizes for ANOVA were obtained by eta squared (*η_p_*^2^) and were interpreted as *η_p_*^2^ < 0.01 “trivial”, 0.01 to 0.06 “low”, =0.06 to 0.14 “moderate”, and >0.14 “high”. When a significant interaction or main effect was found, the post-hoc tests with Bonferroni correction were used to analyze the pairwise comparisons. The magnitude of mean differences was expressed with standardized effect size (ES). Thresholds for qualitative descriptors of Cohen’s d were interpreted as <0.20 “trivial”, 0.2–0.49 “small”, 0.5–0.79 “moderate”, and >0.8 “large” [28]. The independent samples *t*-tests were used to compare participants’ training experience and anthropometrics data. The smallest worthwhile change (SWC, calculated using the formula 0.2 × test values SD) [29] was used to define the responders and non-responders on CA. A participant is considered a responder if the performance improvements exceeded the SWC value, a non-responder if performance change fell within the SWC value, and a negative responder if the performance decreases above SWC. The chi-square test has been performed to identify whether there are differences between responders, non-responders, and negative responders to CA pre- and post-training.

## 4. Results

The Shapiro–Wilk test did not show a statistically significant data distribution violation in any examined variables. In the case of the main effect of the condition for the 10 m sprint and weaker limb peak force, the Greenhouse–Geisser correction has been adopted. No significant differences were reported in age (*p* = 0.208), resistance (*p* = 0.454), or basketball training experience (*p* = 0.631), as well as in body mass (*p* = 0.341) and body height (*p* = 0.241).

### 4.1. Jumping Performance

There were no statistically significant interactions for CMJ height (F = 1.044; *p* = 0.318; *η_p_*^2^ = 0.045), relative peak power (F = 0.008; *p* = 0.93; *η_p_*^2^ = 0.00), contraction time (F = 0.037; *p* = 0.849; *η_p_*^2^ = 0.002), countermovement depth (F = 0.1; *p* = 0.754; *η_p_*^2^ = 0.005). Similarly, there were no main effects of time for CMJ height (F = 2.837; *p* = 0.106; *η_p_*^2^ = 0.114), relative peak power (F = 0.558; *p* = 0.463; *η_p_*^2^ = 0.025), contraction time (F = 0.008; *p* = 0.929; *η_p_*^2^ = 0.000), and countermovement depth (F = 2.207; *p* = 0.152; *η_p_*^2^ = 0.091). Finally, the was no main effect of the group for CMJ height (F = 1.89; *p* = 0.183; *η_p_*^2^ = 0.079), relative peak power (F = 1.101; *p* = 0.305; *η_p_*^2^ = 0.048), contraction time (F = 2.24; *p* = 0.149; *η_p_*^2^ = 0.092), and countermovement depth (F = 1.142; *p* = 0.297; *η_p_*^2^ = 0.049) (Table 3).

There were no statistically significant interactions for dominant SLJ height (F = 0.002; *p* = 0.961; *η_p_*^2^ = 0.00) and relative peak power (F = 0.131; *p* = 0.721; *η_p_*^2^ = 0.006), or for non-dominant SLJ height (F = 0.702; *p* = 0.411; *η_p_*^2^ = 0.031) and relative peak power (F = 0.199; *p* = 0.66; *η_p_*^2^ = 0.009). Similarly, there were no main effects of a group for dominant SLJ height (F = 0.14; *p* = 0.712; *η_p_*^2^ = 0.006) and relative peak power (F = 0.388; *p* = 0.54; *η_p_*^2^ = 0.017), or for non-dominant SLJ height (F = 0.459; *p* = 0.505; *η_p_*^2^ = 0.02), and relative peak power (F = 0.008; *p* = 0.93; *η_p_*^2^ = 0.00). Moreover, there were no statistically significant main effects of time for dominant SLJ height (F = 2.774; *p* = 0.110; *η_p_*^2^ = 0.112). However, there were statistically significant main effects of time to increase dominant SLJ relative peak power (F = 16.55; *p* = 0.001; *η_p_*^2^ = 0.429), non-dominant SLJ height (F = 6.036; *p* = 0.022; *η_p_*^2^ = 0.215), and non-dominant SLJ relative peak power (F = 6.368; *p* = 0.019; *η_p_*^2^ = 0.224) post-training compared to pre (Table 4).

### 4.2. Shuttle Run Test Time

**Figure 2:**
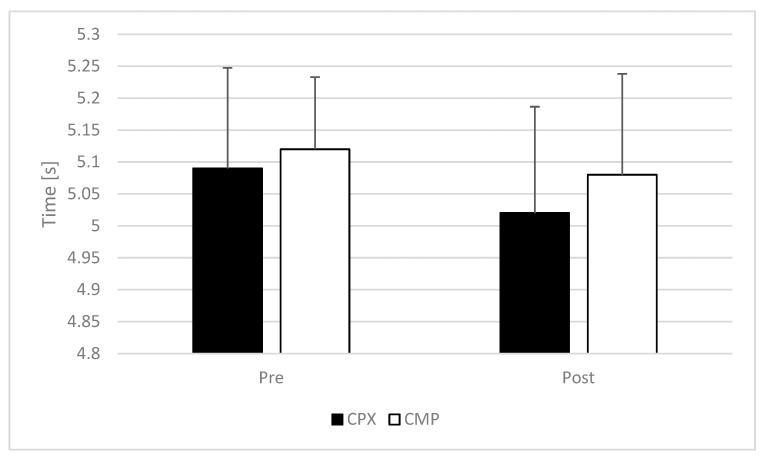
Shuttle run test time comparison between complex and compound resistance training group. CPX—complex training group; CMP—compound resistance training group.

Two-way ANOVA indicated no statistically significant interaction (F = 0.206; *p* = 0.654; *η_p_*^2^ = 0.009)—nor the main effect of time (F = 3.816; *p* = 0.064; *η_p_*^2^ = 0.148)—and a main effect of group (F = 0.829; *p* = 0.372; *η_p_*^2^ = 0.036) for shuttle run test time (Figure 2).

### 4.3. Post-Activation Performance Enhancement and Its Magnitude 

Interactions and main effects of PAPE responses for studied variables are described in Table 5, while descriptive data are presented in Table 6.

The CA was significantly and similarly effective in eliciting PAPE response before and after each intervention (Table 6).

The chi-square test indicated that there were not any significant differences in responders, non-responders, and negative responders between pre-training and post-training for both CPX and CMP groups in CMJ height (*p* = 0.565 and *p* = 0.357, respectively), dominant SLJ height (*p* = 0.627 and *p* = 0.356, respectively), non-dominant SLJ height (*p* = 0.693 and *p* = 0.176, respectively) and shuttle run test time (*p* = 0.264 and *p* = 0.871, respectively). (Table 7).

## 5. Discussion

The aim of this investigation was to compare the effects of CPX and CMP training on CMJ and SLJ performance, shuttle test time, and the magnitude of the PAPE effect in basketball players. The main finding of this study was that both types of training resulted in a statistically significant improvement in SLJ power output for both the dominant and non-dominant legs, as well as the height of the non-dominant SLJ. No statistically significant changes were observed in the other tests, such as CMJ and shuttle test time; however, the observed changes were positive with a trivial to small effect size. Furthermore, neither of the training programs had a significant impact on the magnitude of the PAPE response as well as on responders’ distribution. Nevertheless, it should be noted that the drop jumps performed in this study (as a CA: three sets of five drop jumps) immediately improved the assessed jumping performance and shuttle test time. This study suggests that both CPX and CMP training for 8 weeks significantly and equally improved SLJs, with no significant changes in CMJ and shuttle test time.

The results of the current study are partially consistent with previous findings, which did not demonstrate the superiority of CPX training over traditional resistance training methods in improving jumping and running capabilities [3,12,14,15]. The short-term 4-week protocol by Mihalik et al. [15], the 6-week protocol by Ali et al. [14], our 8-week protocol, and the 10-week protocol by Gee et al. [12] did not show differences between the training programs. However, the mentioned studies demonstrated a significant improvement in the vertical jump; in our study, it was observed only in the single-leg vertical jump but not in the CMJ. The reason for the different results could be related to differences in the training programs, particularly in training volume. Mihalik et al. [15] compared 4-week training programs for the lower limbs, specifically CPX training versus resistance and plyometric training (on separate days). The participants performed three sets of three pairs of exercises twice a week; thus, there are 36 sets per week (144 sets in the whole intervention). In contrast, the CPX training in Gee et al.’s [12] study consisted of three sets of two pairs of exercises twice a week (a total of 24 sets per week, 240 sets in total) involving the lower limbs over 10 weeks. Meanwhile, participants in the Ali et al. [14] study trained three times a week for six weeks with a CPX training program consisting of four pairs of exercises with three sets each (72 sets per week, 432 sets in total). In contrast, in the current study, it was 18 sets (in weeks 1–2) and 24 sets (in weeks 3–6), and then 12 sets (in weeks 7–8), totaling 156 sets, which is significantly fewer than in studies by Gee et al. [12] and Ali et al. [14], and is comparable to Mihalik et al.’s [15] study, but over twice the duration (4 weeks vs. 8 weeks in this study). Considering all of the above, it appears that the volume used in this study might have been insufficient to trigger significant enhancements in CMJ and shuttle test performance. However, it was adequate to improve SLJ performance. Taking both of these outcomes into account, it could be assumed that the most likely reason for the improvement in SLJ performance is the enhancement in participants’ balance rather than an augmentation in the explosive capabilities of their lower limbs. Nevertheless, since no balance assessments were conducted in this investigation, these are only speculations and should be taken into account by future research.

Interestingly, the aforementioned outcomes align partially with the findings of the study by Gee et al. [12]. The authors also did not observe improvement in another change of direction test: the arrowhead changes of direction speed test. In contrast, Ali et al. [14] did not include a change of direction assessment in the test battery in their study, but they included a 20 m sprint test and also did not observe a significant improvement. It can be speculated that the reason lies in the selection of exercises that were not in accordance with the principle of dynamic correspondence [30]. However, despite the fact that only our training included the change of direction running as a post-activation exercise, as well as hip thrusts and squats, which have been shown to effectively improve sprint time in previous studies, no significant changes were observed in the shuttle test time. The trivial transfer of strength training to change-of-direction and sprint performance has also been indicated in earlier studies [31,32,33]. This may be related to the different ranges of motion in which force is produced during resistance exercises compared to running and changes of direction. Furthermore, the fact that the shuttle test used as part of the training did not contribute to a significant improvement in the time achieved after the training intervention may again be related to insufficient volume (only three sets per week). An alternative explanation for the lack of significant improvement in CMJ and shuttle test time, while observing improvement in SLJ, could be explained by the principle of diminishing returns. This principle refers to the decreasing expected degree of improvement in fitness as individuals become fitter [34]. It is possible that the individual level of performance in the CMJ and shuttle test among the participants in this study was already high enough that the observed improvement in these tests did not reach statistical significance. Conversely, they had a noticeable deficit in SLJ, which was successfully improved.

It is worth mentioning that the applied CA in the form of three sets of five drop jumps improved the height of the CMJ, dominant and non-dominant SLJs, as well as shuttle test time. This is another study that confirms the effectiveness of plyometric exercises as CA in inducing the PAPE effect [35,36,37,38]. However, interestingly, in contrast to Dello Iacono et al. [36], the CA used in this study improved both jump height and shuttle test time. Therefore, the result of this study challenges the theory that CA should be force-vector specific and favor the principle of dynamic correspondence. The force-vector theory contends that horizontal exercises are more specific to horizontal sports skills, while vertical exercises transfer better to vertical sports skills. However, studies by Bielitzki et al. [39] and Yetter and Moir [40] showed that a squat-based CA contributed to acute sprint improvement. As Fitzpatrick pointed out, the forces acting on an athlete or expressed by the athlete should be considered with respect to the athlete’s local (established by the athlete) coordinate system, rather than the global coordinate system [30]. So, it seems that instead of the force-vector theory, PAPE complexes should be designed based on the principle of dynamic correspondence, especially the ranges of motion in which force is produced during CA and post-activation tasks.

One aspect that significantly distinguished our study from previous studies was the inclusion of active rest intervals to maintain a high training density in our CPX training protocol. The use of a rest period between the CA and the subsequent movement activity to achieve the PAPE effect is often highlighted as a drawback of this method [12]. In fact, recommended rest periods of 4–8 min significantly decrease the time efficiency of the training, making it time consuming [7]. However, incorporating exercises involving a different body region allowed us to maintain the same time efficiency as in traditional training. Despite this, the training remained equally as effective as our resistance training approach. Therefore, it seems that both types of training can be applied at the discretion of the coach.

Although training experience and training background have been indicated as mediators of the PAPE response [8,19], according to the authors’ knowledge, this is the only study to date that has assessed it. The outcomes of this investigation indicated that neither CPX nor CMP training had an impact on the magnitude of the PAPE response. This suggests that regular implementation of CPX training does not affect the PAPE effect. It is possible that the 8-week training period was not sufficient to significantly impact the PAPE response in any way. In contrast, in both training protocols, participants performed the same high-intensity exercises, with the only difference being the order of execution. Therefore, further research is needed to evaluate factors such as the impact of different training intensities or types on the PAPE response.

The findings of this study should be considered in light of its limitations. Although the training programs implemented in this study included both upper and lower body training, the tests used only assessed the performance of the lower limbs. Moreover, we did not conduct a mid-evaluation of 1 RM, potentially leading to inadequate loading in the later weeks of training, which may not have corresponded to participants’ current strength levels. Furthermore, during the last two weeks, the training volume tapered down, which could have also impacted the outcomes. Although participants were instructed to exert maximal effort during training, we did not assess this using resources such as linear encoder or force plates (in the case of isometric exercises). It is also important to consider that participants were engaged in basketball training during the intervention, thus potential interactions between these methods and their impact on the results should be taken into account. Furthermore, the applied CA for evaluating the changes in the PAPE response was a plyometric exercise, while high-intensity resistance exercises were used as the CA during the intervention. Therefore, it is uncertain how the PAPE response would have changed if the CA had been consistent with the training protocol.

## 6. Conclusions

The results of this study showed that 8 weeks of both CPX and CMP training led to statistically significant improvements in the SLJ power of both the dominant and non-dominant limbs as well as the height of the non-dominant SLJ. However, the magnitude of improvement in CMJ and shuttle test time was trivial to small and did not reach statistical significance. Furthermore, neither of the training methods had a significant impact on the magnitude of the PAPE response or on the responders’ distribution. It is worth noting that the CA applied in this study, consisting of three sets of five drop jumps, immediately improved performance in jumps and shuttle test time. The results of this study suggest that both evaluated training approaches can be used to improve jumping and shuttle test times, but it is suggested to consider a slightly higher training volume.

## Figures and Tables

**Table 1:** Exercise selection and loading parameters for a complex training program.

Workout A								
	Set [n]			Rep [n]/Time [s]	Intensity (1 RM/RPE)			Rest [s]
	Week 1–2	Week 3–6	Week 7–8	Week 1–8	Week 1–2	Week 3–6	Week 7–8	
A1 Back Squat	3	4	2	5	80%	85%	85%	90–120
A2 Face Pulls	3	4	2	8–10	7 RPE	8 RPE	8 RPE	90–120
A3 Drop Jump	3	4	2	5	BM			90–120
B1 Bench Press	3	4	2	5	80%	85%	85%	90–120
B2 Leg Curls	3	4	2	8–10	7 RPE	8 RPE	8 RPE	90–120
B3 Plyometric Push-Ups	3	4	2	5	BM			90–120
C DB Row	2	3	2	10–12	7 RPE	8 RPE	8 RPE	90–120
**Workout B**								
A1 Isometric-Push Split Squat	3	4	2	3 s (each leg)	Maximum Effort			90–120
A2 Plank	3	4	2	30 s	BM			90–120
A3 Shuttle test run	3	4	2	2 (1 on each turn)	Maximum Effort			90–120
B1 Pull-ups	3	4	2	2 RIR	BM			90–120
B2 Hip Thrusts	3	4	2	8–10	7 RPE	8 RPE	8 RPE	90–120
B3 Med Ball Slams	3	4	2	5	5–8 kg			90–120
C Single Arm DB Overhead Press	3	4	2	10–12	7 RPE	8 RPE	8 RPE	90–120

Rep—repetitions; 1 RM—one repetition maximum; RPE—a rate of perceived exertion; BM—body mass; DB—dumbbell; RIR—repetition in reserve.

**Table 2:** Exercise selection and loading parameters of compound resistance training program.

Workout A								
	Set [n]			Rep [n]/Time [s]	Intensity (1 RM/RPE)			Rest [s]
	Week 1–2	Week 3–6	Week 7–8	Week 1–8	Week 1–2	Week 3–6	Week 7–8	
A1 Drop jump	3	4	2	5	BM			90–120
A2 Plyometric Push-Ups	3	4	2	5	BM			90–120
B Back Squat	3	4	2	5	80%	85%	85%	90–120
C1 DB Row	2	3	2	10–12	7 RPE	8 RPE	8 RPE	90–120
C2 Leg Curls	3	4	2	8–10	7 RPE	8 RPE	8 RPE	90–120
D1 Bench Press	3	4	2	5	80%	85%	85%	90–120
D2 Face Pulls	3	4	2	8–10	7 RPE	8 RPE	8 RPE	90–120
**Workout B**								
A1 Shuttle test run	3	4	2	2 (1 on each turn)	Maximum Effort			90–120
A2 Med Ball Slams	3	4	2	5	5–8 kg			90–120
B Hip Thrusts	3	4	2	8–10	7 RPE	8 RPE	8 RPE	90–120
C1 Isometric-Push Split Squat	3	4	2	3 s (each leg)	Maximum Effort			90–120
C2 Pull-ups	3	4	2	2 RIR	BM			90–120
D1 Single Arm DB Overhead Press	3	4	2	10–12	7 RPE	8 RPE	8 RPE	90–120
D2 Plank	3	4	2	30 s	BM			90–120

Rep—repetitions; 1 RM—one repetition maximum; RPE—a rate of perceived exertion; BM—body mass; DB—dumbbell; RIR—repetition in reserve.

**Table 3:** Countermovement jump performance comparison between complex and compound resistance training group.

Performance Type	Group	Pre-Training (95% CI)	Post Training (95% CI)	ES (Interpretation)	|∆|	%∆
CMJ Height [cm]	CPX	35.8 ± 2.5 (34.4 to 37.3)	36.7 ± 3.5 (34.8 to 38.6)	0.3 (small)	0.9 ± 1.8	2.3 ± 5
	CMP	37.7 ± 2.6 (36.2 to 39.3)	37.9 ± 2.9 (35.9 to 40)	0.07 (trivial)	0.2 ± 1.1	0.5 ± 3
CMJ Relative Peak Power [W/kg]	CPX	57.2 ± 4.1 (55.1 to 59.2)	57.7 ± 6.7 (54.5 to 60.9)	0.09 (trivial)	0.5 ± 4.8	0.8 ± 9.1
	CMP	57.7 ± 6.7 (56.7 to 61.2)	59.6 ± 3.7 (56.1 to 63)	0.35 (small)	0.7 ± 2	1.1 ± 3.3
CMJ Contraction Time [ms]	CPX	755 ± 188 (664 to 846)	758 ± 145 (684 to 833)	0.02 (trivial)	4 ± 211	7.3 ± 37
	CMP	833 ± 111 (734 to 932)	823 ± 109 (742 to 904)	0.09 (trivial)	−10 ± 107	−0.5 ± 12.3
CMJ Countermovement Depth [cm]	CPX	22.9 ± 9.2 (18.5 to 27.2)	19.6 ± 9.2 (15 to 24.2)	0.36 (small)	−3.2 ± 11.6	−4.1 ± 64.2
	CMP	25.1 ± 4.7 (20.4 to 29.8)	23 ± 6.3 (18 to 28)	0.38 (small)	−2.1 ± 2.8	−9.5 ± 13.1

CI—confidence interval; ES—effect size; CMJ—countermovement jump; CPX—complex training group; CMP—compound resistance training group.

**Table 4:** Single-leg countermovement jump performance comparison between complex and compound resistance training group.

Performance Type	Group	Pre-Training (95% CI)	Post Training (95% CI)	ES (Interpretation)	|∆|	%∆
DOM SLJ Height [cm]	CPX	16.7 ± 2.9 (14.9 to 18.5)	17.4 ± 3.2 (15.5 to 19.4)	0.23 (small)	0.8 ± 2.7	5.4 ± 16.2
	CMP	17.1 ± 3.3 (15.2 to 19.1)	17.9 ± 3.5 (15.9 to 20)	0.24 (small)	0.8 ± 1.6	5.1 ± 9.9
DOM SLJ Relative Peak Power [W/kg]	CPX	33.2 ± 4.6 (30.6 to 35.8)	34.6 ± 5.1 * (31.7 to 37.4)	0.29 (small)	1.3 ± 2.2	4.1 ± 6.6
	CMP	34.3 ± 4.4 (31.5 to 37.1)	35.9 ± 4.7 * (32.8 to 38.9)	0.35 (small)	1.6 ± 1.1	4.7 ± 3.4
N-DOM SLJ Height [cm]	CPX	16.6 ± 2.6 (15.4 to 17.8)	17.4 ± 2.8 * (16.1 to 18.7)	0.30 (small)	0.8 ± 0.7	4.7 ± 4.5
	CMP	16.2 ± 1.4 (14.9 to 17.6)	16.6 ± 1.5 * (15.2 to 18)	0.28 (small)	0.4 ± 1.5	2.7 ± 9.2
N-DOM SLJ Relative Peak Power [W/kg]	CPX	33.7 ± 3.9 (31.8 to 35.6)	34.5 ± 4.6 * (32.3 to 36.6)	0.19 (trivial)	0.8 ± 1.5	2.2 ± 4.6
	CMP	33.6 ± 2.4 (31.6 to 35.7)	34.7 ± 2.7 * (32.3 to 37.1)	0.43 (small)	1.1 ± 2.1	3.4 ± 6.4

CI—confidence interval; ES—effect size; DOM—dominant limb; N-DOM—non-dominant limb; SLJ—single leg jump; CPX—complex training group; CMP—compound resistance training group; *—significant difference in comparison to pre-training.

**Table 5:** Interactions and main effects of post-activation performance enhancement responses in all tasks.

Variable	Group × Pre/Post-CA × Pre/Post-Training	Group × Pre/Post-CA	Group × Pre/Post-Training	Pre/Post-CA × Pre/Post-Training	Group	Pre/Post-CA	Pre/Post-Training
CMJ height [cm]	F = 0.164; *p* = 0.689; *η_p_*^2^ = 0.007	F = 0.403; *p* = 0.532; *η_p_*^2^ = 0.018	F = 1.039; *p* = 0.319; *η_p_*^2^ = 0.007	F = 1.927; *p* = 0.179; *η_p_*^2^ = 0.081	F = 1.561; *p* = 0.225; *η_p_*^2^ = 0.066	F = 21.715; *p* < 0.001; *η_p_*^2^ = 0.497 *	F = 0.3; *p* = 0.589; *η_p_*^2^ = 0.013
DOM SLJ Height [cm]	F = 0.012; *p* = 0.914; *η_p_*^2^ = 0.001	F = 0.132; *p* = 0.72; *η_p_*^2^ = 0.006	F = 0.00; *p* = 0.989; *η_p_*^2^ = 0.00	F = 1.463; *p* = 0.239; *η_p_*^2^ = 0.062	F = 0.092; *p* = 0.765; *η_p_*^2^ = 0.004	F = 4.793; *p* = 0.039; *η_p_*^2^ = 0.179 *	F = 13.4; *p* = 0.001; *η_p_*^2^ = 0.379 *
N-DOM SLJ Height [cm]	F = 0.225; *p* = 0.64; *η_p_*^2^ = 0.01	F = 0.00; *p* = 0.987; *η_p_*^2^ = 0.00	F = 0.18; *p* = 0.676; *η_p_*^2^ = 0.008	F = 0.166; *p* = 0.688; *η_p_*^2^ = 0.007	F = 0.444; *p* = 0.512; *η_p_*^2^ = 0.02	F = 5.043; *p* = 0.035; *η_p_*^2^ = 0.186 *	F = 5.542; *p* = 0.028; *η_p_*^2^ = 0.201 *
Shuttle Run Test Time [s]	F = 0.807; *p* = 0.379; *η_p_*^2^ = 0.035	F = 0.188; *p* = 0.669; *η_p_*^2^ = 0.008	F = 0.934; *p* = 0.344; *η_p_*^2^ = 0.041	F = 0.387; *p* = 0.54; *η_p_*^2^ = 0.017	F = 0.904; *p* = 0.352; *η_p_*^2^ = 0.039	F = 16.447; *p* = 0.001; *η_p_*^2^ = 0.428 *	F = 7.693; *p* = 0.011; *η_p_*^2^ = 0.259 *

CA—conditioning activity; CMJ—countermovement jump; DOM—dominant limb; N-DOM—non-dominant limb; SLJ—single leg jump; *—statistically significant.

**Table 6:** Comparison of post-activation performance enhancement responses in all tasks.

Performance Type	Group	Pre-Training				Post Training			
		Pre-CA (95%CI)	Post-CA (95%CI)	ES	%∆	Pre-CA (95%CI)	Post-CA (95%CI)	ES	%∆
CMJ Height [cm]	CPX	35.8 ± 2.5 (34.3–37.3)	37.8 ± 3.5 * (35.7 to 39.9)	0.66	5.5 ± 6.7	36.7 ± 3.5 (34.6 to 38.8)	38.2 ± 2.9 * (36.5 to 39.9)	0.47	4.5 ± 5
	CMP	37.7 ± 2.6 (36 to 39.4)	39.5 ± 3 * (37.4 to 41.5)	0.64	4.6 ± 3.8	37.9 ± 2.9 (36 to 39.9)	38.9 ± 3 * (36.9 to 40.9)	0.34	2.6 ± 5.3
DOM SLJ Height [cm]	CPX	16.7 ± 2.9 (15 to 18.4)	17.1 ± 3 * (15.2 to 18.9)	0.14	2.3 ± 5.6	17.5 ± 3.2 (15.5 to 19.4)	18.5 ± 2.6 * (16.9 to 20.1)	0.34	7.6 ± 14.8
	CMP	17.1 ± 3.3 (14.9 to 19.4)	17.4 ± 3.1 * (15.3 to 19.4)	0.09	1.7 ± 6.4	17.9 ± 3.5 (15.6 to 20.3)	18.7 ± 3.3 * (16.5 to 20.9)	0.24	5.4 ± 14.6
N-DOM SLJ Height [cm]	CPX	16.6 ± 2.6 (15 to 18.2)	17.0 ± 3.1 * (15.1 to 18.8)	0.14	2.3 ± 6.3	17.4 ± 2.8 (15.7 to 19.1)	17.8 ± 3.2 * (15.8 to 19.7)	0.13	2 ± 7.7
	CMP	16.2 ± 1.4 (15.2 to 17.2)	16.4 ± 0.9 * (15.8 to 17)	0.17	1.9 ± 6.7	16.6 ± 1.5 (15.6 to 17.6)	17.1 ± 1.5 * (16.1 to 18.1)	0.33	3.7 ± 6.2
Shuttle Run Test Time [s]	CPX	5.09 ± 0.16 (5.01 to 5.17)	5.06 ± 0.09 * (5.01 to 5.12)	0.23	−0.7 ± 1.3	5.02 ± 0.17 (4.92 to 5.11)	4.96 ± 0.15 * (4.89 to 5.04)	0.37	−1 ± −0.9
	CMP	5.12 ± 0.11 (5.04 to 5.21)	5.07 ± 0.09 * (5.02 to 5.13)	0.5	−0.8 ± 1.4	5.08 ± 0.16 (4.98 to 5.18)	5.03 ± 0.11 * (4.95 to 5.12)	0.36	−0.7 ± 1.4

CA—conditioning activity; ES—effect size; CMJ—countermovement jump; CPX—complex training group; CMP—compound resistance training group; DOM—dominant limb; N-DOM—non-dominant limb; SLJ—single leg jump; *—a statistically significant difference in comparison to Pre-CA in corresponding time point.

**Table 7:** Smallest worthwhile change values and distribution of responders, non-responders, and negative responders on applied conditioning activity.

Performance Type	Group	SWC		Responders [n]		Non-Responders [n]		Negative Responders [n]	
		Pre-Training	Post-Training	Pre-Training	Post-Training	Pre-Training	Post-Training	Pre-Training	Post-Training
CMJ Height [cm]	CPX	0.5	0.7	9	9	3	4	1	0
	CMP	0.51	0.59	9	8	1	0	1	3
DOM SLJ Height [cm]	CPX	0.57	0.63	7	8	4	2	2	3
	CMP	0.67	0.7	4	5	5	2	2	4
N-DOM SLJ Height [cm]	CPX	0.52	0.55	6	4	2	2	5	7
	CMP	0.29	0.31	7	5	4	3	0	3
Shuttle Run Test Time [s]	CPX	0.03	0.03	9	9	2	4	2	0
	CMP	0.02	0.03	7	6	2	3	2	2

CMJ—countermovement jump; CPX—complex training group; CMP—compound resistance training group; DOM—dominant limb; N-DOM—non-dominant limb; SLJ—single leg jump.

## Data Availability

Data is available on request from the corresponding author.
